# An integrative multi-omic analysis defines gut microbiota, mycobiota, and metabolic fingerprints in ulcerative colitis patients

**DOI:** 10.3389/fcimb.2024.1366192

**Published:** 2024-05-08

**Authors:** Matteo Scanu, Francesca Toto, Valentina Petito, Letizia Masi, Marco Fidaleo, Pierluigi Puca, Valerio Baldelli, Sofia Reddel, Pamela Vernocchi, Giovambattista Pani, Lorenza Putignani, Franco Scaldaferri, Federica Del Chierico

**Affiliations:** ^1^ Immunology, Rheumatology and Infectious Diseases Research Area, Unit of Human Microbiome, Bambino Gesù Children’s Hospital, IRCCS, Rome, Italy; ^2^ Dipartimento di Scienze Mediche e Chirurgiche, Unità Operativa Semplice di Malattie Infiammatorie Croniche Intestinali, CEMAD, Unità Operativa Complessa di Medicina Interna e Gastroenterologia, Fondazione Policlinico Universitario Agostino Gemelli IRCCS, Rome, Italy; ^3^ Department of Biology and Biotechnologies "Charles Darwin", Sapienza University of Rome, Rome, Italy; ^4^ CNIS Research Center for Nanotechnology Applied to Engineering, Sapienza University of Rome, Rome, Italy; ^5^ Dipartimento di Medicina e Chirurgia Traslazionale, Università Cattolica del Sacro Cuore, Rome, Italy; ^6^ Unit of Microbiology and Diagnostic Immunology, Unit of Microbiomics and Research Area of Immunology, Rheumatology and Infectious Diseases, Unit of Human Microbiome, Bambino Gesù Children’s Hospital, IRCCS, Rome, Italy

**Keywords:** inflammatory bowel disease, ulcerative colitis, dysbiosis, gut microbiota, gut metabolism, intestinal biomarkers, multi-omic integrated approaches

## Abstract

**Background:**

Ulcerative colitis (UC) is a multifactorial chronic inflammatory bowel disease (IBD) that affects the large intestine with superficial mucosal inflammation. A dysbiotic gut microbial profile has been associated with UC. Our study aimed to characterize the UC gut bacterial, fungal, and metabolic fingerprints by omic approaches.

**Methods:**

The 16S rRNA- and ITS2-based metataxonomics and gas chromatography–mass spectrometry/solid phase microextraction (GC–MS/SPME) metabolomic analysis were performed on stool samples of 53 UC patients and 37 healthy subjects (CTRL). Univariate and multivariate approaches were applied to separated and integrated omic data, to define microbiota, mycobiota, and metabolic signatures in UC. The interaction between gut bacteria and fungi was investigated by network analysis.

**Results:**

In the UC cohort, we reported the increase of *Streptococcus*, *Bifidobacterium*, Enterobacteriaceae, TM7-3, *Granulicatella*, *Peptostreptococcus*, *Lactobacillus*, *Veillonella*, *Enterococcus*, *Peptoniphilus*, Gemellaceae, and phenylethyl alcohol; and we also reported the decrease of *Akkermansia*; Ruminococcaceae; *Ruminococcus*; *Gemmiger*; *Methanobrevibacter*; *Oscillospira*; *Coprococus*; *Christensenellaceae*; *Clavispora*; *Vishniacozyma*; *Quambalaria*; hexadecane; cyclopentadecane; 5-hepten-2-ol, 6 methyl; 3-carene; caryophyllene; p-Cresol; 2-butenal; indole, 3-methyl-; 6-methyl-3,5-heptadiene-2-one; 5-octadecene; and 5-hepten-2-one, 6 methyl. The integration of the multi-omic data confirmed the presence of a distinctive bacterial, fungal, and metabolic fingerprint in UC gut microbiota. Moreover, the network analysis highlighted bacterial and fungal synergistic and/or divergent interkingdom interactions.

**Conclusion:**

In this study, we identified intestinal bacterial, fungal, and metabolic UC-associated biomarkers. Furthermore, evidence on the relationships between bacterial and fungal ecosystems provides a comprehensive perspective on intestinal dysbiosis and ecological interactions between microorganisms in the framework of UC.

## 1 Introduction

The human gut microbiota, mycobiota, metabolome, and their interactions contribute to gastrointestinal (GI) health and immune system homeostasis (Strati et al., 2021). An alteration in the composition or function of the intestinal microbiota establishes a dysbiotic state of the gut (Sovran et al., 2018; Lee et al., 2022), which is associated with several human diseases, including inflammatory bowel diseases (IBDs) (Lee et al., 2022).

IBDs are multifactorial diseases whose etiopathogenesis resulted from the complex interactions among immune system dysregulation, genetic and environmental factors, and intestinal homeostasis disorders (Wijmenga, 2005; Knights et al., 2014). Based on disease manifestation, IBD is classified into two major subtypes: ulcerative colitis (UC) and Crohn’s disease (CD) (Xavier and Podolsky, 2007). In particular, UC, the most common form of IBD, affects the large intestine (colon and rectum) with mucosal inflammation that can lead to complications (i.e., ulceration, severe bleeding, toxic megacolon, and fulminant colitis) (Chang, 2020). UC is associated with several risk genic loci (Anderson et al., 2011) involved in epithelial barrier dysfunction, apoptosis and autophagy, and transcriptional and adaptive immune dysregulation (Danese and Fiocchi, 2011). CD results in transmural ulceration of any portion of the GI, most often affecting the terminal ileum and colon (Xavier and Podolsky, 2007).

Both CD and UC are characterized by chronic inflammation of the GI tract, caused by an abnormal immune response to a dysbiotic gut microbiota marked by an overgrowth of harmful bacteria and concomitant depletion of beneficial members (McDowell et al., 2023). This dysbiotic condition plays a pivotal role in the triggering and maintenance of intestinal inflammatory processes in these diseases (Bryan et al., 2016; Zheng and Wen, 2021; Wiredu Ocansey et al., 2023).

Moreover, it is noteworthy to consider the effects of the changes in the composition of intestinal mycobiota in these patients. Some studies have reported low levels of *Saccharomyces cerevisiae* and high levels of *Candida albicans* in UC patients compared with healthy subjects (Sokol et al., 2017; Imai et al., 2019; Chen et al., 2022). Furthermore, the Basidiomycota/Ascomycota ratio was high in UC patients during flares but normal in remission, suggesting their involvement in the inflammatory processes (Sokol et al., 2017; Imai et al., 2019; Chen et al., 2022).

As a result of microbiota and mycobiota dysbiosis, broad changes in gut microbial metabolism have been reported in IBD patients with dysbiosis (Heinken et al., 2021). For example, alterations in fecal bile acids (BAs) and in inflammatory responses have been demonstrated in UC patients as a result of the dysregulation of the gut microbiota (Gallagher et al., 2021; Sultan et al., 2021; Yang et al., 2021). Furthermore, the increase of fecal amino acids in UC has been correlated with intestinal dysbiosis and malabsorption caused by persistent intestinal inflammation (Marchesi et al., 2007).

There are several studies that have dealt with defining the role of the intestinal microbiota in IBDs with single omics approaches, while there are still few integrated omics studies that offer a holistic point of view on this topic.

The purpose of this study was to define the gut bacterial, fungal, and metabolomic profiles of UC patients, by an innovative and complete biocomputational approach. Moreover, by the integration of these omic profiles, we targeted the identification of disease-associated biomarkers. Finally, by the ecological interkingdom connection study, we aimed to establish synergistic and/or divergent interactions between bacteria and fungi and their role in intestinal dysbiosis.

## 2 Materials and methods

### 2.1 Patients and samples

In this study, patients in the active stage of UC, according to the Mayo clinical score, were enrolled at the Internal Medicine and Gastroenterology Division at Fondazione Policlinico Universitario “A. Gemelli” IRCCS Hospital.

The inclusion criteria were as follows: diagnosis of UC, mild to moderate active disease (Mayo clinical score, MCS ≤4) or moderate to severe active disease >4, naive to biologic therapies or having failed no more than one line of biologic treatment, and candidate to second-generation therapies. The exclusion criteria were the presence of infectious, ischemic, and actinic colitis or other significant comorbidities.

Healthy subjects, selected for gender and age matching with UC patients, were enrolled at the Human Microbiome Unit of Bambino Gesù Children’s Hospital in Rome, during an epidemiological survey. Subjects with a family history of autoimmune or IBD diseases, with gastrointestinal diseases, and using either antibiotics or pre-/probiotics in the previous 2 months from enrollment were excluded. Fecal samples were collected and stored to generate a reference sample biobank of healthy subjects (BBMRI Human Microbiome Biobank, OPBG).

This study was approved by the Ethics Committee of Fondazione Policlinico Universitario “A. Gemelli” IRCCS Hospital (Protocol 25062019 n.884) and of Bambino Gesù Children’s Hospital, IRCCS (healthy subjects: No. 1113_OPBG_2016), and was conducted in accordance with the Principles of Good Clinical Practice and the Declaration of Helsinki. All participants provided written informed consent for participation in this study.

### 2.2 16S rRNA and ITS2 loci sequencing

For bacterial metagenomic analysis, 200 mg of stools were submitted to DNA extraction by QIAmp Fast DNA Stool mini kit (Qiagen, Germany), according to the manufacturer’s instructions.

A 16S rRNA gene fragment, comprising the V3 and V4 hypervariable regions, was amplified using primers reported in the MiSeq rRNA Amplicon Sequencing protocol (Illumina, San Diego, CA, USA).

The approach used for fungal metagenomic analysis started with the lysis step obtained by the incubation of 200 mg of stools resuspended in 500 μl of lysis solution (50 mM of Tris [pH 7.5], 10 mM of EDTA, 28 mM of 2-mercaptoethanol, 10 U/ml of lyticase) (Merck KGaA, Darmstadt, Germany) at 37°C for 30 min in agitation at 850 rpm. After the lysis step, DNA extraction was obtained as described above.

The ITS2 region of approximately 350 bp was amplified using the primers ITS2 5′-GTGARTCATCGAATCTTT-3′ and 5′-GATATGCTTAAGTTCAGCGGGT-3′ (Lemoinne et al., 2020; Del Chierico et al., 2024) under the following conditions: 94°C for 2 min, 35 cycles of 15 s at 94°C, 52°C for 30 s, and 72°C for 45 s. The PCR products were purified using AMPure XP Beads (Beckman Coulters, Brea, CA, USA). A second step of PCR was performed with Illumina-adapted ITS2 primers: 5′-TCGTCGGCAGCGTCAGATGTGTATAAGAGACAGGTGARTCATCGAATCTTT-3′ and 5′-GTCTCGTGGGCTCGGAGATGTGTATAAGAGACAGATATGCTTAAGTTCAGCGGGT-3′, following the previously reported PCR conditions applied for 15 cycles.

After a second step of DNA purification, an amplicon PCR indexing step was performed (Nextera XT Index Kit, Illumina) (Del Chierico et al., 2024).

Both bacterial and fungal final libraries were quantified by Quant-iT™ PicoGreen^®^ dsDNA assay kit (Thermo Fisher Scientific, Waltham, MA, USA), pooled, and sequenced on an Illumina MiSeq™ platform, according to the manufacturer’s specifications. For all amplification steps, negative and positive controls were used to exclude eventual internal and external contaminations for both 16S rRNA and ITS2 sequencing approaches.

All raw sequencing reads are available at the NCBI BioProject database (PRJNA996768 and PRJNA996917) (https://submit.ncbi.nlm.nih.gov/subs/sra/).

### 2.3 16S rRNA and ITS2 data analyses

Bioinformatics analysis was performed by QIIME2 v.2022.2 software (Bolyen et al., 2019), using DADA2 (Callahan et al., 2016) plugin for quality check, trimming of forward and reverse fastq files, denoising, chimera filtering, and merging reads. The representative sequences of each amplicon sequence variant (ASV) produced with a cutoff of 99% similarity were annotated by using a naive Bayes classifier against the Greengenes reference database (v13.8, http://www.greengenes.secondgenome.com) (DeSantis et al., 2006) for bacteria and the UNITE ITS dynamic database (v9.0, https://unite.ut.ee) (Nilsson et al., 2019) for fungi.

Statistical analyses were performed with R software v4.0.4. For α- and β-diversity analyses, rarefaction was applied on the feature table with absolute frequency, filtering out 16,140 and 2,870 ASVs for 16S and ITS2, respectively. Statistical analyses on α-diversity indices were performed using the non-parametric Mann–Whitney and Kruskal–Wallis tests. The PERMANOVA test was applied to β-diversity matrices. For further analyses, a raw feature table was normalized with the cumulative sum scaling (CSS) method (Paulson et al., 2013).

### 2.4 Metabolomic profiling determination

To characterize and quantify volatile organic compounds (VOCs), 119 stool samples were analyzed with gas chromatography (GC) combined with mass spectrometry (MS) coupled with solid-phase microextraction (SPME) (Douny et al., 2019). The carboxen-polydimethylsiloxane-coated fiber (CAR-PDMS) (85 μm) and the manual solid-phase microextraction (SPME) (Supelco Inc., Bellefonte, PA, USA) were exposed to each sample, for 45 min. The latter was then inserted into the GC injection port for the desorption phase of the samples for 10 min, and GC–MS analyses were carried out using the Agilent Technologies 7890B GC coupled to a 5977A mass selective detector by operating in electron impact mode, equipped with an Agilent DB-HeavyWaX (60 m length, 0.25 mm ID, 0.25 µm) capillary column. All processes were performed under the same conditions reported by Vernocchi et al. (2020).

A match probability of 80%, or greater, was used for VOC identification followed by manual visual inspection of the fragment patterns when necessary. Furthermore, 4-methyl-2-pentanol (final concentration, 400 ppm) was used as an internal standard (IS) in all analyses to quantify the compounds via interpolation of the relative areas in comparison to the IS area (expressed as mg/kg).

VOCs were identified by using a two-step process: the peak spectrum was tested against the NIST (NIST version 2005, NIST 14MS database; National Institute of Standards and Technology, Rockville, MD, USA) mass spectral library database and literature (Garner et al., 2007), and thereafter, in case further confirmation is needed for the NIST identification, it was confirmed by comparing the retention times of the peaks of interest vs. retention times obtained for the reference standards.

### 2.5 Statistical analysis and omic data integration

ASVs (bacteria: present less than 25% of THE total samples and with relative abundance <0.01; fungi: present less than 25% of the total samples) and metabolites (present less than 10% of the total samples) were filtered out.

Metadata distribution was analyzed by the Shapiro–Wilk test. Gender, age, and clinical features (i.e., corticosteroid therapy, previous therapy, and failure to previous therapies) were evaluated as confounding factors by microbiomeMarker v1.6.0 R package. Age was tested further with the PERMANOVA test by adonis2 function of Vegan v2.6 R package and by linear discriminant analysis (LDA) effect size (LEfSe) (Segata et al., 2011) to exclude it as a confounding factor.

To evaluate the differences in the microbiota, mycobiota, and metabolic profiles between the control (CTRL) and UC patient groups, univariate and multivariate approaches have been applied: linear discriminant analysis effect size, principal component analysis (PCA), and partial least squares-discriminant analysis (PLS-DA). Bacteria function profile was predicted with the Phylogenetic Investigation of Communities by Reconstruction of Unobserved States of Correlation 2 (PICRUSt2) software (Douglas et al., 2020) on 16S rRNA metagenomic data. The *p-*values were corrected by the Benjamini and Hochberg method to control the false discovery rate (FDR). In all statistical analyses, differences with an adjusted *p-*value <0.05 were considered significant.

Correlation networks between bacterial and fungal communities were built using Spearman’s correlation by means of the graph and corrr R packages (v1.74.0 and v0.4.4, respectively).

PCA was applied to bacterial, fungal, and metabolite matrices. Then, differential -omic features were screened using variable importance in the projection (VIP) values >1 of the first two principal components of the PLS-DA model and compared with those obtained by differential log fold change of univariate analysis by the Mann–Whitney *U* test.

The integration of multiple omic data was performed with multivariate approaches: unsupervised ComDim (Common Dimension) multiblock method (Qannari et al., 2000), unsupervised multiblock principal component analysis (MBPCA) (Tchandao Mangamana et al., 2019), and supervised multiblock partial least squares-discriminant analysis (MBPLS-DA) (Brandolini-Bunlon et al., 2019). Each omic matrix (data blocks) was normalized with Frobenius’s method, to harmonize concentration values of metabolites with the relative abundance of microorganisms (Curtasu et al., 2020). Finally, all matrices were joined into a final unique matrix to perform multivariate analyses. MBPLS-DA was validated with the area under the receiver operating characteristic (AUROC) curve, RMSE, *Q* ^2^, and *R* ^2^ values.

## 3 Results

### 3.1 Characteristics of the overall cohort

In this observational study, 53 patients with UC and 37 healthy subjects (CTRL) were enrolled. The cohort characteristics are reported in **Table 1**.

**Table 1:** Characteristics of the UC and CTRL groups.

Characteristics	UC (*n* = 53)	CTRL (*n* = 37)
Gender, *N* (%)		
**Male**	25 (47.2%)	17 (46%)
**Female**	28 (52.8%)	20 (54%)
Age (years)		
**Mean ± SD**	40.47 ± 14.14	50.70 ± 14.80
Disease duration (years)		
**Mean ± SD**	8.83 ± 7.19	–
Clinical features		
**Corticosteroid therapy, *N* (%)**	41 (77.3%)	–
**Previous therapy, *N* (%)**	21 (39.6%)	–
**Failure to previous therapies, *N* (%)**	18 (33.9%)	–

SD, standard deviation.

### 3.2 Gut microbiota was independent from gender, age, and clinical characteristics

By metataxonomic analysis of fecal samples, we obtained 10,729 bacterial and 1,484 fungal ASVs.

We tested gender, age, and some clinical variables as confounding factors in our analyses. No differences in gut microbiota taxa and metabolite distribution were observed for gender, corticosteroid therapy, previous therapy, and failure of previous therapy (**Supplementary Table 1**). On the contrary, age resulted as a confounding factor for gut microbiota analysis (*p-*value = 0.032). Then, we performed the beta-diversity analysis on the microbial, fungal, and metabolic variables of subjects grouped by median age (46 years) by the Bray–Curtis algorithm. The PERMANOVA test applied to the dissimilarity matrix showed no statistical significance between the two groups (*p-*value > 0.05) (**Supplementary Figure 1**). This result was also confirmed by the LEfSe univariate analysis that revealed no differences in bacterial, fungal, and metabolomic distribution by median age (FDR > 0.05) (**Supplementary Table 2**).

### 3.3 Gut bacterial and fungal dysbiosis in UC

Comparing UC and CTRL, the α-diversity analysis, calculated on the bacterial composition, revealed no statistically significant differences between UC and CTRL (**Supplementary Figure 2A**), while a significant decrease of the Shannon–Wiener index, calculated on the fungal dataset, was found (*p-*value = 0.01) (**Supplementary Figure 2B**).

The β-diversity analysis based on the Bray–Curtis algorithm revealed two distinct clusters for UC and CTRL (PERMANOVA *p-*value < 0.05) (**Supplementary Figures 2C, D**) and an increase of intragroup distance in UC than in CTRL (*p-*value = 0.0001), for both bacterial and fungal ecosystems (**Supplementary Figures 2E, F**).

**Figure 1:**
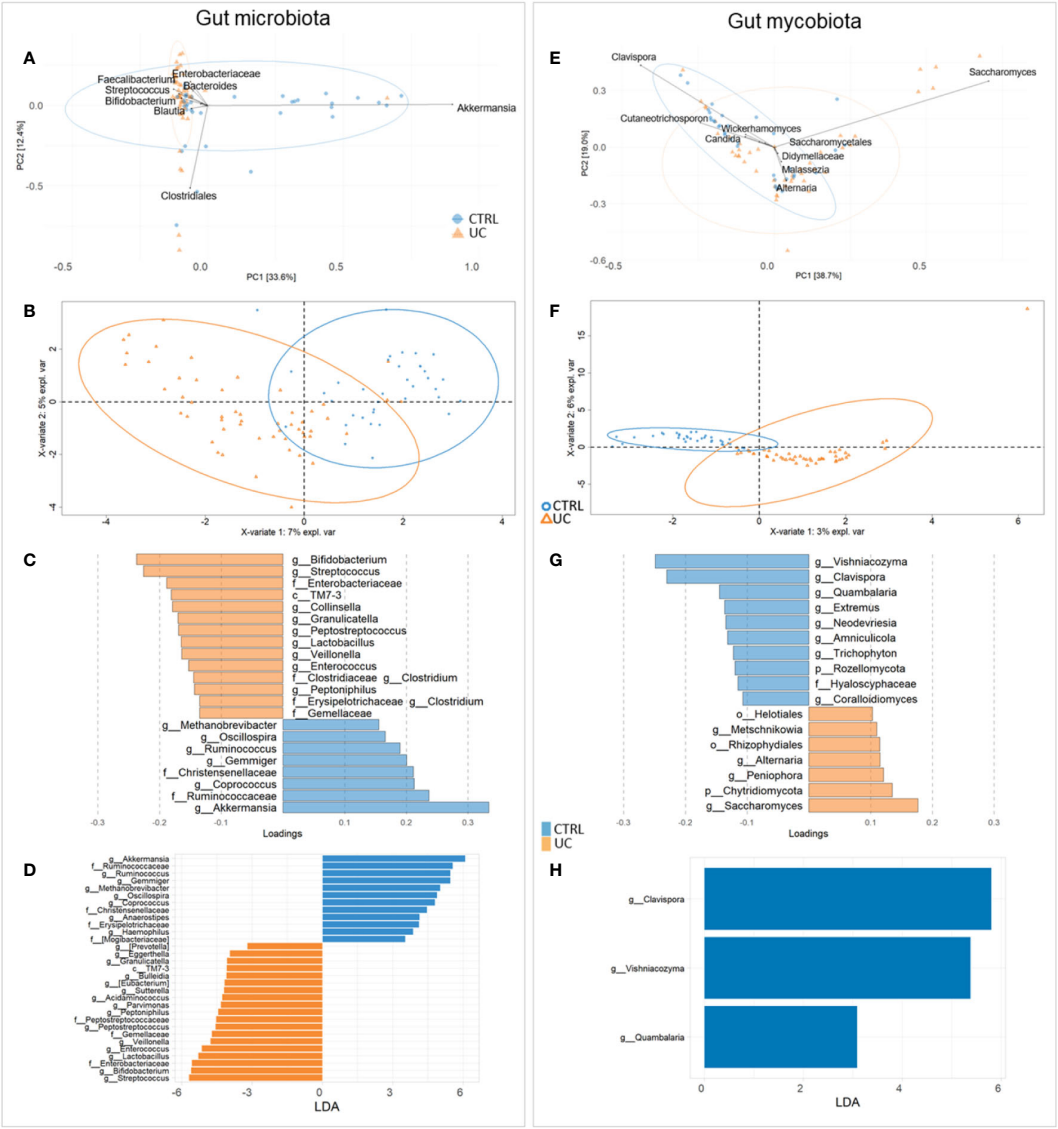
Compositional analysis at the genus level of UC and CTRL gut microbiota (left panel) and mycobiota (right panel). Unsupervised multivariate analysis [principal component analysis (PCA) plot] **(A, E)**; supervised multivariate analysis plot [partial least squares-discriminant analysis (PLS-DA)] **(B, F)** and loading variables plot (filtered for VIP > 1 and for fungi, for loading coefficient > 0.1) **(C, G)**. Bacterial PLS-DA is characterized by root mean square error (RMSE) = 0.336, *R* ^2^ value = 0.544, and *Q* ^2 ^= 0.418. Fungal PLS-DA is characterized by RMSE = 0.226, *R* ^2^ = 0.761, and *Q* ^2^ value = 0.168. LDA plots on LEfSe univariate analysis **(D, H)**. Bacterial taxa enriched in UC patients have negative LDA scores (orange), while bacterial and fungal taxa enriched in CTRL have positive scores (blue).

To investigate the differences in gut microbiota and mycobiota composition between UC and CTRL, we applied multivariate and univariate approaches (**Figure 1**).

By the fusion of these results, we assigned *Streptococcus*, *Bifidobacterium*, Enterobacteriaceae, TM7-3, *Granulicatella*, *Peptostreptococcus*, *Lactobacillus*, *Veillonella*, *Enterococcus*, *Peptoniphilus*, and Gemellaceae to UC and *Akkermansia*, Ruminococcaceae, *Ruminococcus*, *Gemmiger*, *Methanobrevibacter*, *Oscillospira*, *Coprococus*, and Christensenellaceae to CTRL gut microbiota (**Figures 1A–D**).

For fungi, PCA identified Didymellaceae, Saccharomycetales, *Malassezia*, *Wickerhamomyces*, *Cutaneotrichosporon*, *Saccharomyces*, *Clavispora*, *Alternaria*, and *Candida* as major fungal markers (**Figure 1E**). PLS-DA analysis revealed the distinctive fecal fungal markers associated with UC (**Figures 1F, G**). Univariate analysis showed a predominance of fungal markers such as *Clavispora*, *Vishniacozyma*, and *Quambalaria* in the CTRL group compared to the UC group, confirming a remarkable difference in the fecal mycobiota between the two groups (**Figure 1H**).

The area under the ROC curve (AUROC) was 0.9393 for bacteria and 0.9951 for fungi, indicating that the applied models have high accuracy in group classifications (**Supplementary Figures 3**, **4**, respectively).

The global composition of the gut microbiota of the study cohorts, represented by the distribution of the ASVs at the phylum, family, and genus levels, is shown in **Supplementary Figure 5**. Compared with healthy subjects, the following bacterial markers were more representative in the microbiota of UC patients: Actinobacteria, Proteobacteria, and Bacteroidetes at the phylum level (**Supplementary Figure 5A**); Ruminococcaceae, Enterobacteriaceae, Bifidobacteriaceae, and Streptococcaceae at the family level (**Supplementary Figure 5B**); and *Streptococcus*, *Faecalibacterium*, *Bifidobacterium*, and *Bacteroides* at the genus level (**Supplementary Figure 5C**). Furthermore, the fungi Ascomycota, Chytridiomycota (**Supplementary Figure 5D**), Saccharomycetaceae, Pleosporaceae, Didymellaceae (**Supplementary Figure 5E**), and *Saccharomyces*, *Malassezia*, and *Alternaria* (**Supplementary Figure 5F**) were the main components of the gut mycobiota in UC patients.

To assess the microbial metabolic pathways, inferred by 16S rRNA sequences, we performed the prediction of pathways of the two cohorts, shown in the LDA plot (**Supplementary Figure 6**). Twenty pathways, belonging to nine defined metabolic classes and one undefined one, have been associated with the UC profile. Of these, the following pathways were increased in UC: amino acid biosynthesis, aspartate superpathway, carbohydrate degradation, enzyme cofactor biosynthesis, fermentation to lactate, fermentation to lactate/acetate, generation of precursor metabolites and energy, generation of precursor metabolites and energy, purine nucleotide biosynthesis, purine nucleotide *de-novo* biosynthesis, and terpenoid biosynthesis.

### 3.4 Bacterial and fungal interkingdom interactions

**Figure 2:**
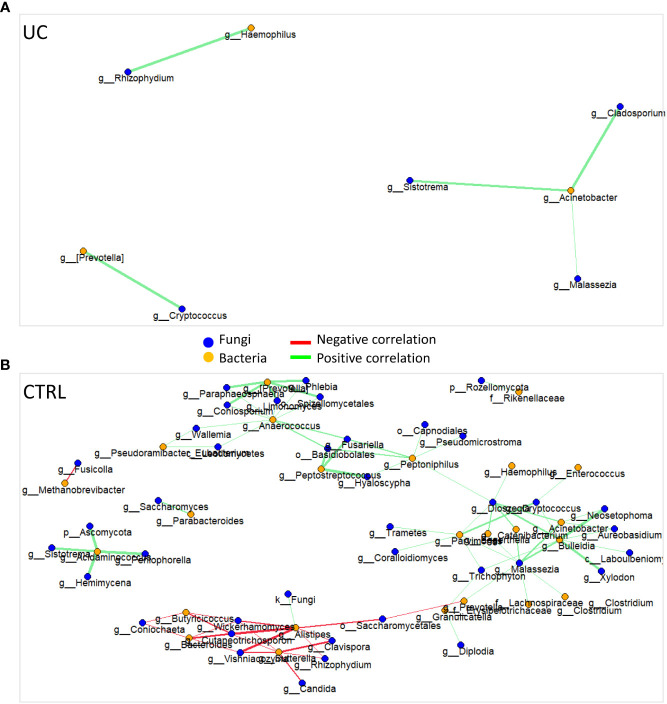
Bacterial and fungal interkingdom correlation network in UC **(A)** and CTRL **(B)**. Each node represents bacteria (orange circles) and fungi (blue circles). Green and red edges indicate positive and negative correlation values, respectively. Only correlations statistically significant (*p*-value < 0.05) are reported.

To gain insight into the relationship between taxa from different kingdoms and to gain a more comprehensive understanding of the microbial ecosystems, we applied the interkingdom correlation network analyses to the bacterial and fungal profiles in both cohorts (**Figures 2A, B**).

The UC cohort network was characterized by eight nodes connected by five edges, a relative connectedness of 0.625, and an average number of neighbors of 2.25 (**Figure 2A**). The CTRL cohort network was characterized by 63 nodes connected by 82 edges, a relative connectedness of 1.30, and an average number of neighbors of 3.6 (**Figure 2B**). All significant correlations between bacteria and fungi are listed in **Supplementary Table 3**.

### 3.5 Distinctive metabolome in UC

**Figure 3:**
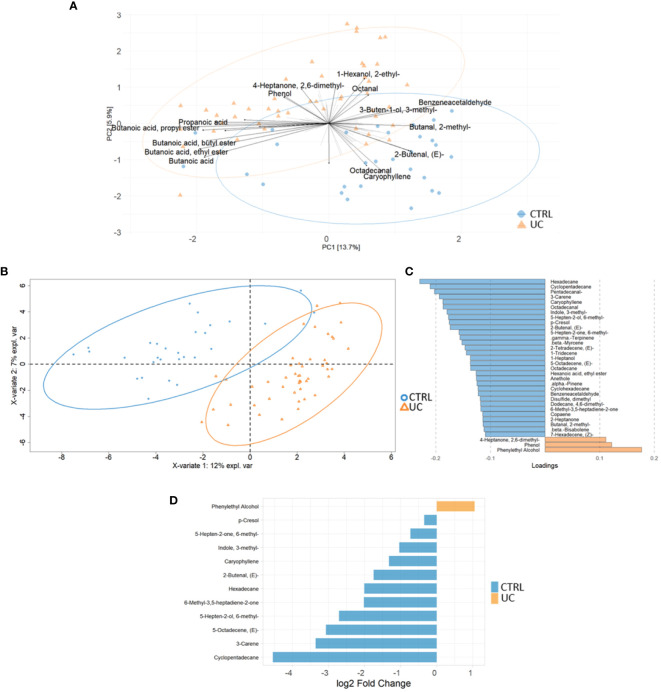
Multivariate and univariate analyses on metabolic profiles of UC and CTRL. The biplot shows the first 24 loadings predicted by PCA analysis **(A)**. The second biplot shows the sample clustering calculated with PLS-DA analysis [blue dots (CTRL) and orange triangles (UC)] **(B)**. The barplot describes the value of loadings in each group, which are calculated by PLS-DA analysis and filtered for loading coefficient >0.1 and VIP value >1 **(C)**. Root mean square error (RMSE) = 0.329, *R* ^2^ = 0.526, and *Q* ^2^ value = 0.382. Univariate plot based on log2 fold change values **(D)**. The Mann–Whitney test confirms that phenylethyl alcohol is increased in the UC group.

We identified 95 filtered molecules by the metabolomic analysis of fecal samples. PCA analysis revealed the presence of two distinct metabolic profiles in UC patients and CTRLs, consisting mainly of 24 metabolic markers (**Figure 3A**).

The PLS-DA analysis showed a low RMSE value (0.329), indicating a high accuracy of the model in predicting the subject classification and highlighting more molecules associated with CTRLs with UC (**Figures 3B, C**). The VIP features are shown in **Supplementary Figure 7A**, and the AUROC was 0.9944 (**Supplementary Figure 7B**). Finally, the univariate analysis confirmed the results reported by the multivariate approaches (**Figure 3D**). The combination of multivariate and univariate test results highlighted the increase of phenylethyl alcohol and the decrease of cyclopentadecane; 5-octadecene; 5-hepten-2-ol, 6 methyl; 6-methyl-3,5-heptadiene-2-one; hexadecane; 2-butenal; caryophyllene; indole, 3-methyl-; 3-carene; p-Cresol; and 5-hepten-2-one, 6-methyl- in UC.

### 3.6 Different integrative multi-omic approaches confirm the presence of a typical shape and function of UC gut microbiota

**Figure 4:**
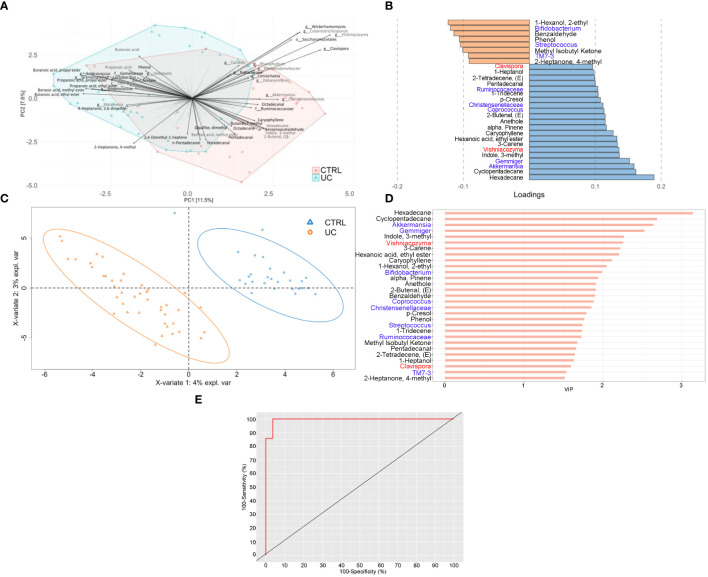
Integrated multi-omic analyses confirm the presence of a typical shape and function of UC gut microbiota. Multiblock principal component analysis (MBPCA) plot **(A)**, loadings plot (filtered for loading coefficient > 0.1) **(B)**, and multiblock partial least squares-discriminant analysis (MBPLS-DA) plot **(C)**. Root mean square error (RMSE) = 0.128, *R* ^2^ = 0.935, and *Q* ^2^ value = 0.567. VIP values are reported on the horizontal axis **(D)**. ROC analysis of the MBPLS-DA model **(E)**. The value of AUROC = 0.9947 indicates a high accuracy of the prediction model.

To reduce the complexity of these multi-omic results, we finally applied three integrative multi-omic approaches to the three omic datasets. The first two were predictive analyses, based on an unsupervised MBPCA and a supervised MBPLS-DA, able to predict discriminant variables (loadings), maintaining multi-omic data separated. The third one was an exploratory approach, carried out by multivariate unsupervised ComDim, in which the three omic matrices were integrated before the analysis. As reported in **Figure 4A**, the MBPCA identified two distinct gut bacterial, fungal, and metabolic profiles in UC and CTRL.

The MBPLS-DA analyses identified loadings for each sample set (**Figures 4B, C**). Regarding UC, the MBPLS-DA revealed an increase of *Bifidobacterium*; *Streptococcus*; TM7-3; 1-hexanol, 2-ethyl-; phenol; benzaldehyde; methyl isobutyl ketone; and 2-heptanone, 4-methyl.

In CTRL, we found the increase of *Akkermansia*; *Gemmiger*; *Coprococcus*; Ruminococcaceae; Christensenellaceae; *Clavispora*; *Vishniacozyma*; cyclopentadecane; 3-carene; 1-tridecene; hexadecane; indole, 3-methyl; hexanoic acid ethyl ester; caryophyllene; alpha-pinene; anethole; 2-butenal; p-Cresol; pentadecanal; 2-tetradecene; and 1-heptanol. The RMSE and *R* ^2^ values were 0.128 and 0.935, respectively, indicating a high performance of this model. The bar plot of VIPs from MBPLS-DA is shown in **Figure 4D**. The ROC analysis (**Figure 4E**) revealed an AUROC value of 0.9947, indicating a high accuracy of the prediction model.

**Figure 5:**
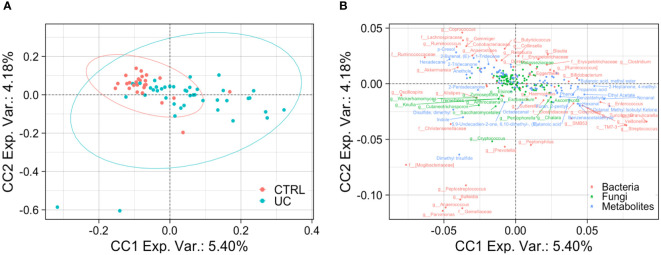
Bacterial, fungal, and metabolic markers in UC and CTRLs. Biplots show the result of ComDim analysis. Teal circles represent the UC patients and red circles represent CTRL subjects **(A)**. Bacterial, fungal, and metabolic markers are labeled in red, green, and blue, respectively **(B)**.

The application of the ComDim analysis on the three omic matrices integrated confirmed the presence of two distinct UC and CTRL profiles, characterized by bacterial, fungal, and metabolic markers (**Figures 5A, B**).

## 4 Discussion

While several studies have investigated the gut microbiota composition in the UC context analyzing omic datasets singularly, we aimed to elucidate, for the first time, the gut bacterial, fungal, and metabolomic profiles of UC patients with innovative biocomputational approaches based on multivariate models applied on separated and integrated omic datasets. With this statistical design, we were able to show a string and well-defined gut microbial and metabolomic fingerprint in UC condition.

Before exploring the gut microbiota composition between UC and CTRL, we excluded age, gender, corticosteroid therapy, previous therapy, and failure to previous therapies as confounders of our analyses.

Consistent with the scientific literature that reports a dysbiotic profile of UC patients with lower gut ecology than CTRL (Xu et al., 2022; Zuo et al., 2022), our results showed a lower UC fungal α-diversity than CTRL, indicating a lower richness and evenness of fungal ecology. This finding was confirmed by the results of the network analysis, in which the UC gut microbiota showed less complexity than those of the CTRL, with a reduced number of intra- and interconnections between bacteria and fungi, probably due to the proinflammatory UC gut microenvironment affecting the microbes and vice versa.

By the combination of multivariate and univariate test results, we showed in UC gut microbiota the increase of *Streptococcus*, *Bifidobacterium*, Enterobacteriaceae, TM7-3, *Granulicatella*, *Peptostreptococcus*, *Lactobacillus*, *Veillonella*, *Enterococcus*, *Peptoniphilus*, and Gemellaceae and the decrease of *Akkermansia*, Ruminococcaceae, *Ruminococcus*, *Gemmiger*, *Methanobrevibacter*, *Oscillospira*, *Coprococus*, and Christensenellaceae. These results agree with other studies describing the composition of the gut microbiota in IBD and specifically in UC, reinforcing the evidence that bacterial intestinal dysbiosis is a strong signature of this disease. Today, microbial biomarkers are proposed for monitoring and evaluating disease activity, predicting recurrence or response to treatment, and treating diseases (Guo et al., 2022; Zheng et al., 2022; Huang et al., 2023).

There is increasing evidence of the relevance of fungal dysbiosis in the pathogenesis of IBD (Sokol et al., 2017; Gu et al., 2019; Balderramo et al., 2023). Fungi can exert direct proinflammatory effects or modify the bacterial composition via interkingdom, opening the possibility of modulating fungal microbiota as a therapeutic approach (Sokol et al., 2017).

In our study, we showed a decrease in *Clavispora*, *Vishniacozyma*, and *Quambalaria* in the CTRL group with UC. *Clavispora*, a member of the *Saccharomyces* genus, exerts a positive effect on the gut by the production of the anti-inflammatory interleukin (IL)-10 (Meng et al., 2022). Moreover, *Clavispora* and *Vishniacozyma* were negatively correlated with *Sutterella* in CTRL, suggesting a negative effect of these two fungi on *Sutterella* growth. Low levels of *Sutterella* in the gut microbiota have been associated with gut immune homeostasis and high levels of IgA, which protect the gut against pathobiont invasion (Kaakoush, 2020). Interestingly, in our study, the UC gut microbiota was enriched with *Sutterella*. This microorganism seems capable of degrading IgA molecules, activating the pattern recognition receptors (PRRs), and producing IL-8, creating a pathological gut microenvironment (Kaakoush, 2020). Furthermore, in the CTRL network, *Sutterella* was also negatively correlated with *Rhizophydium*. We can speculate that fungi could either directly or indirectly reduce *Sutterella* levels, indicating their possible use as probiotics to modulate the presence of bacterial pathobionts in the intestine. Another evidence supporting the influence of fungi on pathobiont increase was represented by the positive correlation between *Rhizophydium* and *Haemophilus* in the UC network. The last, together with *Veillonella*, is known to be associated with disease progression and clinical severity in UC (Basha et al., 2023). Moreover, in our UC cohort, *Cryptococcus* and *Prevotella* were positively correlated, reinforcing the evidence that *Cryptococcus neoformans* and *Prevotella* could contribute to intestinal dysbiosis (Li et al., 2023a). In particular, the genus *Prevotella*, already identified as a UC biomarker (Zois et al., 2010), exhibits enhanced proinflammatory properties, releasing inflammatory mediators and promoting mucosal Th17 immune responses and neutrophil recruitment (Larsen, 2017). In fact, *Prevotella* produces mucin-degrading sulfatases (Wright et al., 2000) and contributes to chronic inflammation by altering the barrier function of epithelial cells in active UC (Tsai et al., 1992, Tsai et al., 1995; Lucke et al., 2006), affecting disease outcomes (Larsen, 2017).

In IBD patients, high levels of fungi with potential proinflammatory effects such as *Candida* and *Malassezia* and low levels of fungi with anti-inflammatory effects such as *Saccharomyces* were reported (Krawczyk et al., 2023). In our study, *Saccharomyces* was effectively present in the CTRL network and positively correlated with *Parabacteroides*. Regarding *Malassezia*, it was present in both networks and was positively correlated with *Acinetobacter*, which is a known IBD biomarker (Yang et al., 2021). *Malassezia* is a lipid-dependent opportunistic basidiomycetous yeast that is capable of epithelial barrier disruption, inflammatory factor accumulation, and proinflammatory cytokine production (Nelson et al., 2021; Balderramo et al., 2023).

These findings suggest that the interaction between gut bacteria and gut fungi is important in the pathology of UC in particular and of IBD in general. However, whether it is bacterial dysbiosis that favors fungal growth or whether it is the expansion of fungal populations that leads to bacterial dysbiosis remains to be fully elucidated.

In terms of metabolic fingerprint, through the combination of multivariate and univariate test results, we showed the increase of phenylethyl alcohol and the decrease of cyclopentadecane; 5-octadecene; 5-hepten-2-ol, 6 methyl; 6-methyl-3,5-heptadiene-2-one; hexadecane; 2-butenal; caryophyllene; indole, 3-methyl; 3-carene; p-Cresol; and 5-hepten-2-one, 6 methyl in the UC gut. Phenylethyl alcohol is produced by fungi such as *C. albicans* (Han et al., 2013) and *Saccharomyces* (Lu et al., 2023) as well as by bacteria such as *Bifidobacterium* (Yan et al., 2022). Interestingly, our results showed high levels of phenylethyl alcohol and its producers in UC. Among the metabolites higher in the CTRL, p-Cresol is a methyl phenol produced via microbial degradation of tyrosine and other aromatic amino acids (Hinai et al., 2019). In our study, we observed that pathways involved in the biosynthesis of aromatic amino acids, such as tyrosine and phenylalanine, were more enriched in the gut microbiota of UC patients than CTRLs, suggesting a negative correlation between the production of p-Cresol and the aromatic amino acid biosynthesis in the gut microbiota of UC patients. However, p-Cresol was also suggested as a biomarker of protein intake (Patel et al., 2012). Surprisingly, indole, 3-methyl-, which is a metabolic product of bacterial tryptophan (Trp) metabolism and is involved in gut dysbiosis, was found to be more abundant in the gut microbiota of CTRLs than in UC patients. However, the occurrence of indole, 3-methyl- in the gut depends on many factors, such as the high intake of Trp-containing proteins, polyphenols, and dietary fiber (Zgarbová and Vrzal, 2023). The combination of polyphenol and fiber fermentation in the colon contributes to a reduction in bacterial populations and an increase in the production of harmful metabolites, including indole, 3-methyl- (Zgarbová and Vrzal, 2023). Furthermore, caryophyllene was also observed to be more abundant in the gut microbiota of CTRLs, and it has been reported in a recent paper as a metabolite with potential benefits in anti-inflammatory responses (Li et al., 2023b). High levels of enones (i.e., 5-hepten-2-ol, 6-methyl-; 6-methyl-3,5-heptadiene-2-one; and 5-hepten-2-one, 6-methyl-) may be related to the host dietary habits rather than specific bacterial metabolism (Escobar Rodríguez et al., 2021). Among the metabolites higher in the CTRL, 3-carene shows anti-inflammatory properties, by slowing down bacterial growth and leading to bacterial metabolic dysfunction and cell membrane disruption (Shu et al., 2019; Wang et al., 2022). In addition, we observed new metabolites that were decreased in the gut microbiota of UC patients, such as cyclopentadecane, 5-octadecene, hexadecane, and 2-butenal, which have not been previously reported in IBD.

Finally, given the high complexity of our large-scale omic datasets, we applied different integrated biostatistical approaches that allowed data dimension reduction, sample clustering, and the association among variables with different numerical scales, useful in UC-associated biomarker prediction. In particular, we applied two unsupervised models (i.e., ComDim and MBPCA) based on an exploratory approach to describe the clustered distribution of UC and CTRL subjects and a supervised model (i.e., MBPLS-DA) to emphasize the most important omic signatures that highlighted the differences between UC and CTRL groups. Compared with the unsupervised models, the MBPLS-DA performed a prediction with prior knowledge of the subjects’ groups and thus showed more clustering of the UC and CTRL groups.

Our results are novel and promising, but there are some limitations in our study. Even if our patient cohort is well homogeneous for clinical features and the CTRL cohort matches for age and gender with patients, the two cohorts were relatively small. Further studies on larger cohorts would undoubtedly reduce the error rate, produce stronger correlations, and validate the compositional and functional gut microbiome profiles characterizing UC.

In conclusion, we are the first to apply both a separate and an integrated omics approach. We have defined a distinctive gut microbiota, mycobiota, and metabolic signature that advances our knowledge of the etiopathogenesis of UC. The multivariate models applied on multi-omic datasets allowed us to have a holistic view of the gut environment in UC. Moreover, we are confident that the proposed statistical approach, based on the coupling of separated and integrated omic datasets, is an innovative way to uncover novel gut microbiota-related biomarkers. Finally, exploiting the gut bacteria and fungi ecological networks provided a comprehensive perspective on intestinal dysbiosis.

## Data availability statement

The datasets presented in this study can be found in online repositories. The names of the repository/repositories and accession number(s) can be found in the article/**Supplementary Material**.

## Ethics statement

The studies involving humans were approved by the Ethics Committee of Fondazione Policlinico Universitario “A. Gemelli” IRCCS Hospital and Bambino Gesù Children’s Hospital, IRCCS. The studies were conducted in accordance with the local legislation and institutional requirements. The participants provided their written informed consent to participate in this study.

## Author contributions

MS: Writing – original draft, Formal analysis, Investigation. FT: Formal analysis, Investigation, Writing – original draft. VP: Investigation, Data curation, Writing – review & editing. LM: Data curation, Investigation, Writing – review & editing. MF: Data curation, Investigation, Writing – review & editing. PP: Data curation, Writing – review & editing. VB: Writing – review & editing, Formal analysis. SR: Formal analysis, Writing – review & editing. PV: Formal analysis, Writing – review & editing. GP: Writing – review & editing, Data curation. LP: Data curation, Writing – review & editing. FS: Writing – review & editing, Conceptualization, Funding acquisition, Resources, Supervision, Visualization. FDC: Conceptualization, Funding acquisition, Resources, Supervision, Visualization, Writing – review & editing, Writing – original draft.
