# Supplementary material for: An integrative multi-omic analysis defines gut microbiota, mycobiota, and metabolic fingerprints in ulcerative colitis patients
Source: Front Cell Infect Microbiol. 2024 May 8;14:1366192. doi: 10.3389/fcimb.2024.1366192 (PMC11109417; doi:10.3389/fcimb.2024.1366192)
Supplement: Supplementary file 1 [file DataSheet_1.docx]

Supplementary Material

# Supplementary Figures and Tables

## Supplementary Figures

**
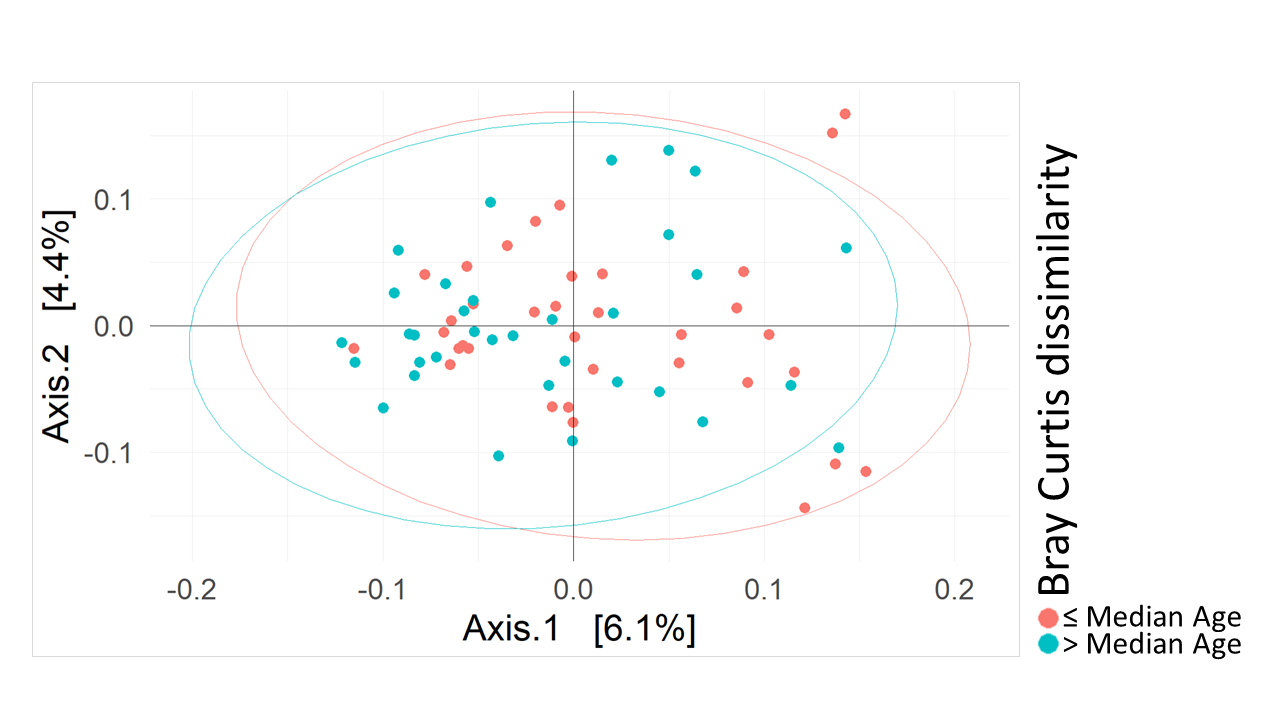
**

**Supplementary Figure 1.** Beta diversity analysis performed of the faecal microbiota, mycobiota and metabolome composition of subjects grouped by median age (46 years) (Bray-Curtis algorithm and presented as a Principal Coordinate Analysis (PCoA) plot). The PERMANOVA test was applied to the β-diversity distance matrix and the result was not statistically significant (*p*-value > 0.05).

**
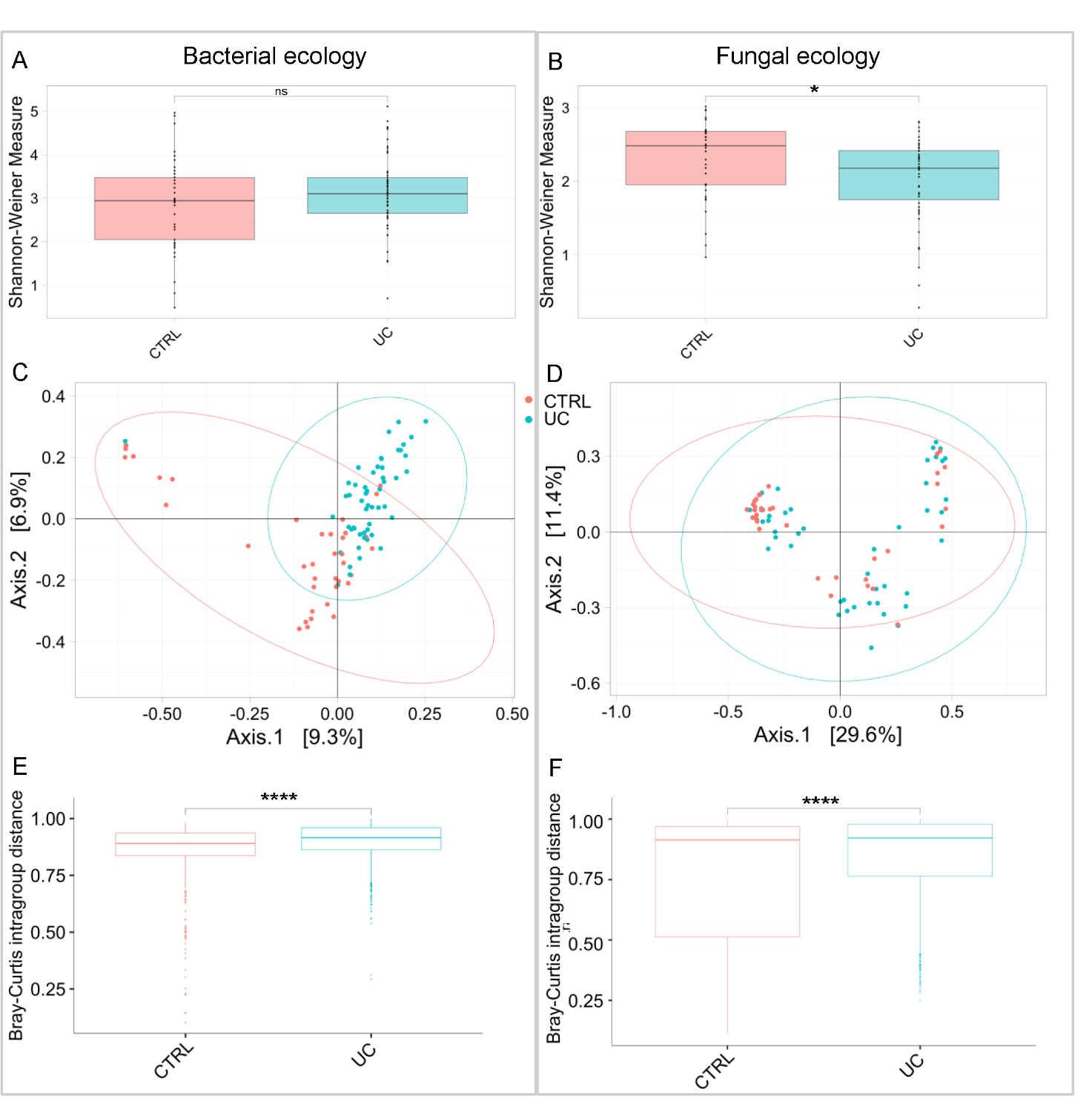
**

**Supplementary Figure 2.** Altered gut microbiota (left panel) and mycobiota (right panel) biodiversity in UC. Alpha diversity, based on the Shannon-Weiner index, was calculated for bacteria **(A)** and fungi **(B)**. The Mann-Whitney test was applied for the comparisons between UC and CTRL. Beta-diversity was calculated by the Bray-Curtis algorithm and presented by PCoA plots for bacteria **(C)** and fungi **(D)**. PERMANOVA test results are statistically significant, *p*-value = 0.001 for bacteria ecology and *p*-value = 0.02 for fungi. Bray Curtis intragroup distance comparisons between UC and CTRL show the increase of intragroup distance in UC, indicating a greater heterogeneity of gut microbiota **(E)** and mycobiota **(F)** composition compared to CTRL. Mann-Whitney tests indicate statistically significant differences (****, *p*-value = 0.0001; *, *p*-value < 0.05; ns, *p*-value > 0.05).


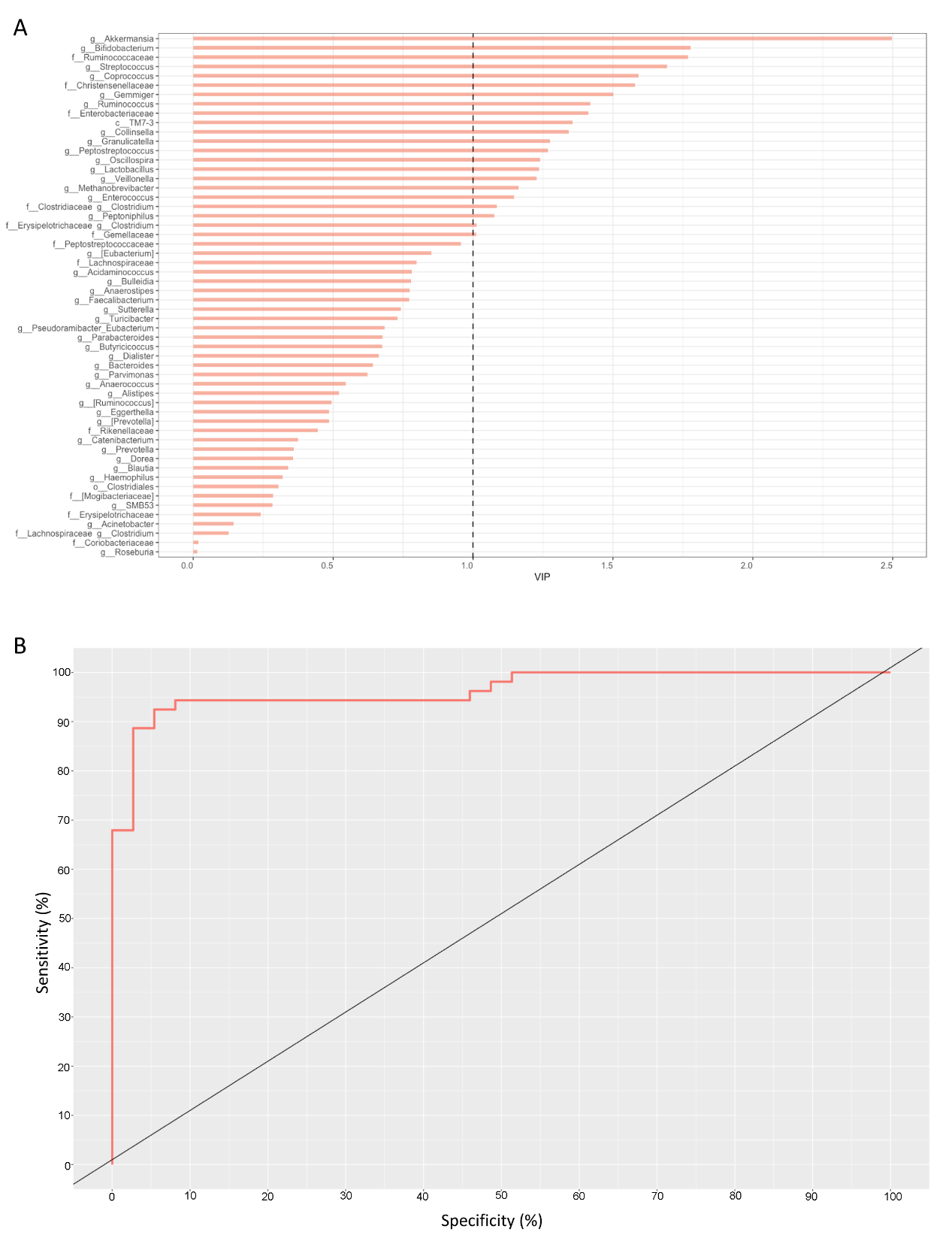


**Supplementary Figure 3.** Multivariate analysis on the bacterial dataset. Bar plot of variable importance in the projection (VIP) from partial least square-discriminant analysis (PLS-DA) **(A)**. VIP values are reported on the horizontal axis. Receiver operating characteristic (ROC) analysis of the PLS-DA model **(B)**. The value of AUROC=0.9393 indicates a high accuracy of the prediction model.

**
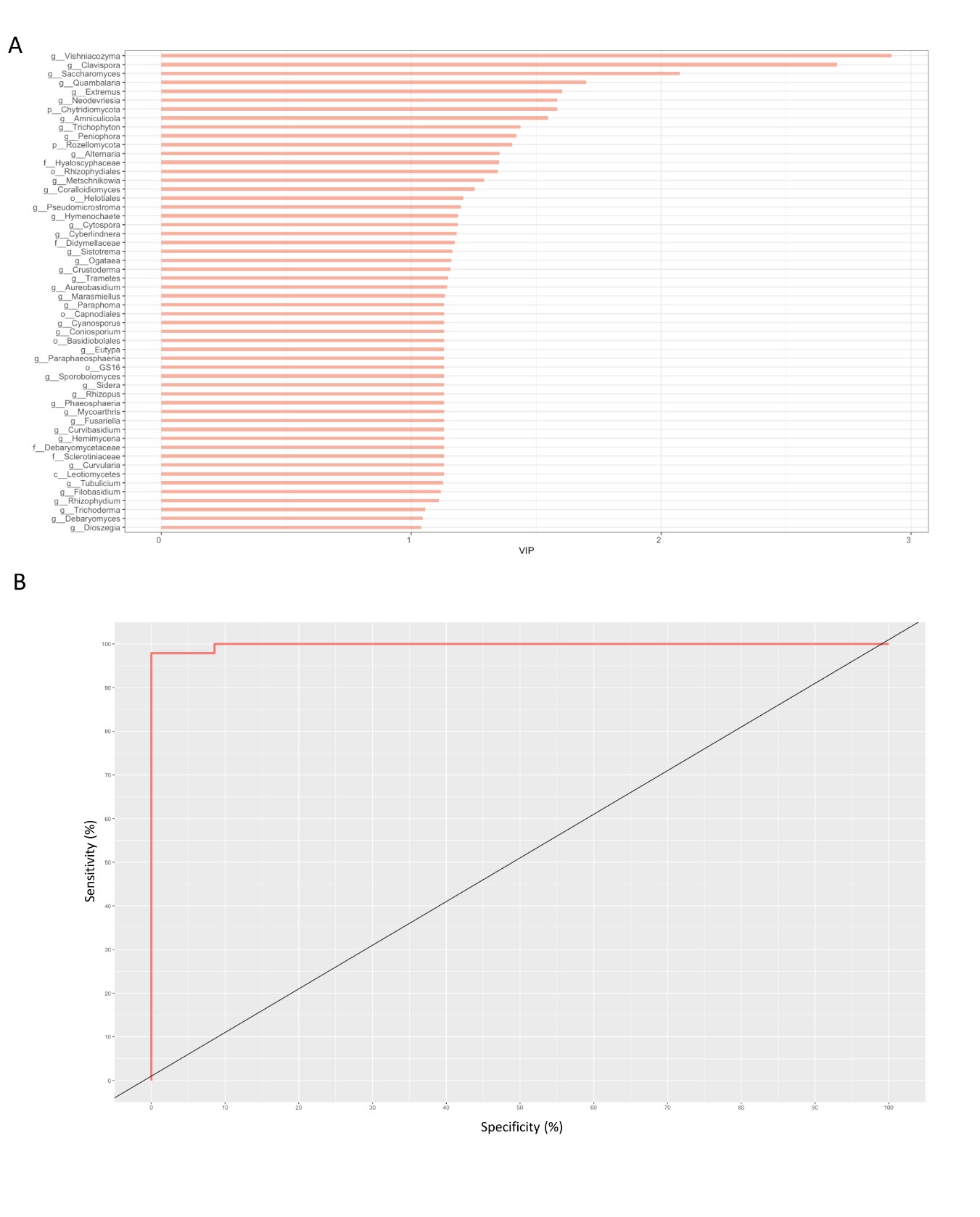
**

**Supplementary Figure 4.** Multivariate analysis on the fungal dataset. Bar plot of VIP from PLS-DA. VIP values are reported on the horizontal axis **(A)**. ROC analysis of the PLS-DA model **(B)**. The value of AUROC=0.9951 indicates a high accuracy of the prediction model.


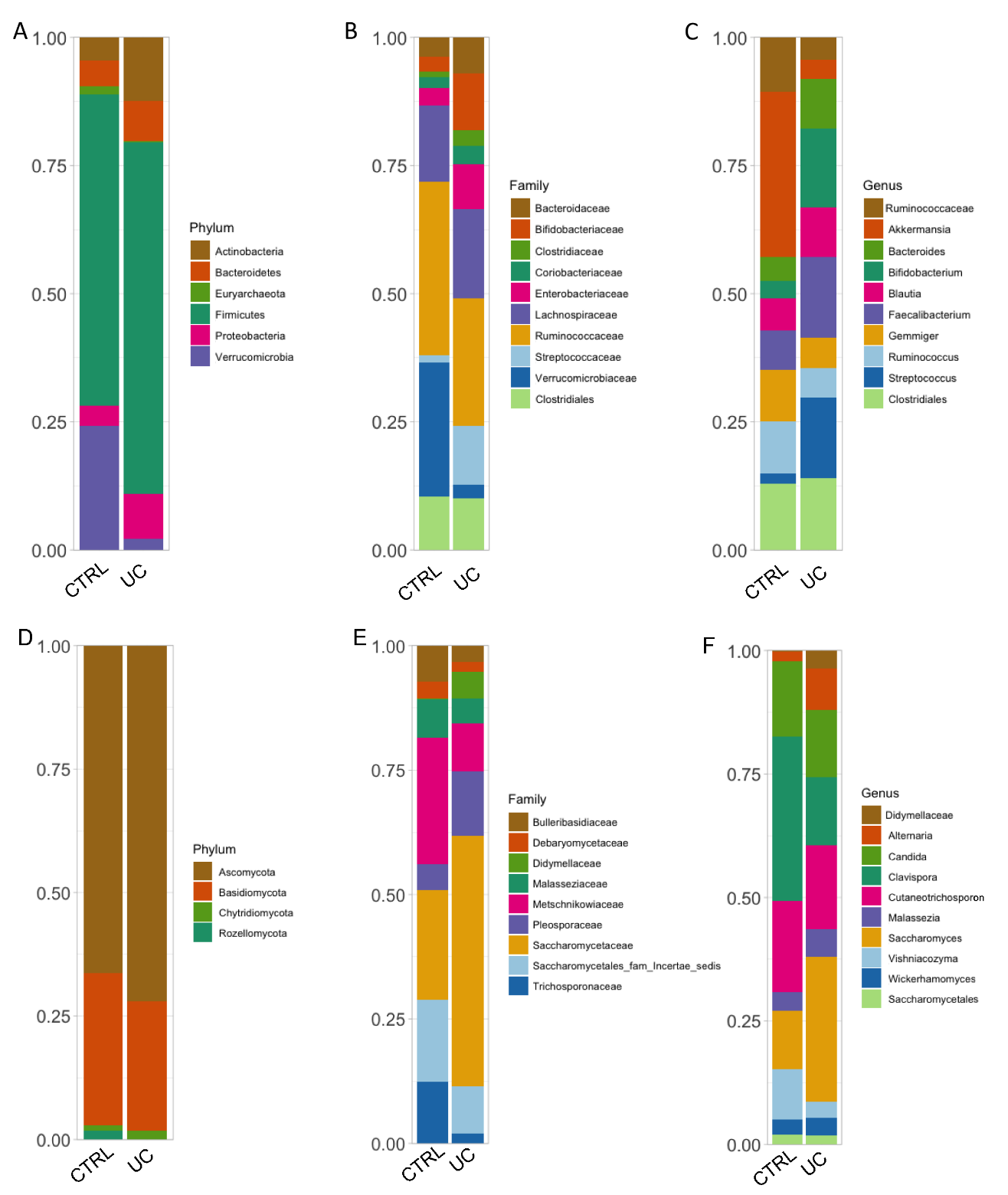


**Supplementary Figure 5.** Distribution of ASVs at phylum, family and genus level in UC and CTRL. Histograms reporting the relative abundance of bacteria in UC and CTRL. At the phylum level, Firmicutes is the predominant phylum in both groups **(A)**. One of the most striking features is the increased abundance of Actinobacteria, Bacteroidetes, Proteobacteria and the decreased abundance of Verrucomicrobia, Euryarchaeota in UC compared to CTRL **(A)**. At family level, higher levels of Bacteroidaceae, Bifidobacteriaceae, Clostridiaceae, Coriobacteriaceae, Enterobacteriaceae and Streptococcaceae and lower abundances of Verrucomicrobiaceae and Ruminococcaceae are observed in UC than CTRL **(B)***.* At the genus level *Streptococcus, Faecalibacterium, Blautia, Bifidobacterium* and *Bacteroides* were more abundant in the UC cohort than in the healthy CTRL group **(C)**. On the other hand, the *genera* *Akkermansia, Ruminococcaceae, Gemmiger* and *Ruminococcus* are less represented in UC than in CTRL **(C)**. Histograms reporting the relative abundance of fungi in UC and CTRL. At the phylum level, Ascomycota, Basidiomycota, Chytridiomycota are increased and Rozellomycota is decreased in the UC cohort **(D)**. At the family level, Saccharomycetaceae, Pleosporaceae, Didymellaceae are increased in the UC cohort, whereas Bulleribasidiaceae, Debaryomycetaceae, Malasseziaceae, Metschnikowiaceae, Saccharomycetales_fam_Incertae_sedis, Trichosporonaceae are decreased **(E)**. At the genus level, *Saccharomyces, Alternaria, Didymellaceae, Malassezia* are increased and *Wicherhamomyces* is slightly increased in the UC cohort, whereas *Clavispora* and *Vishniacozyma* are decreased **(F)**.
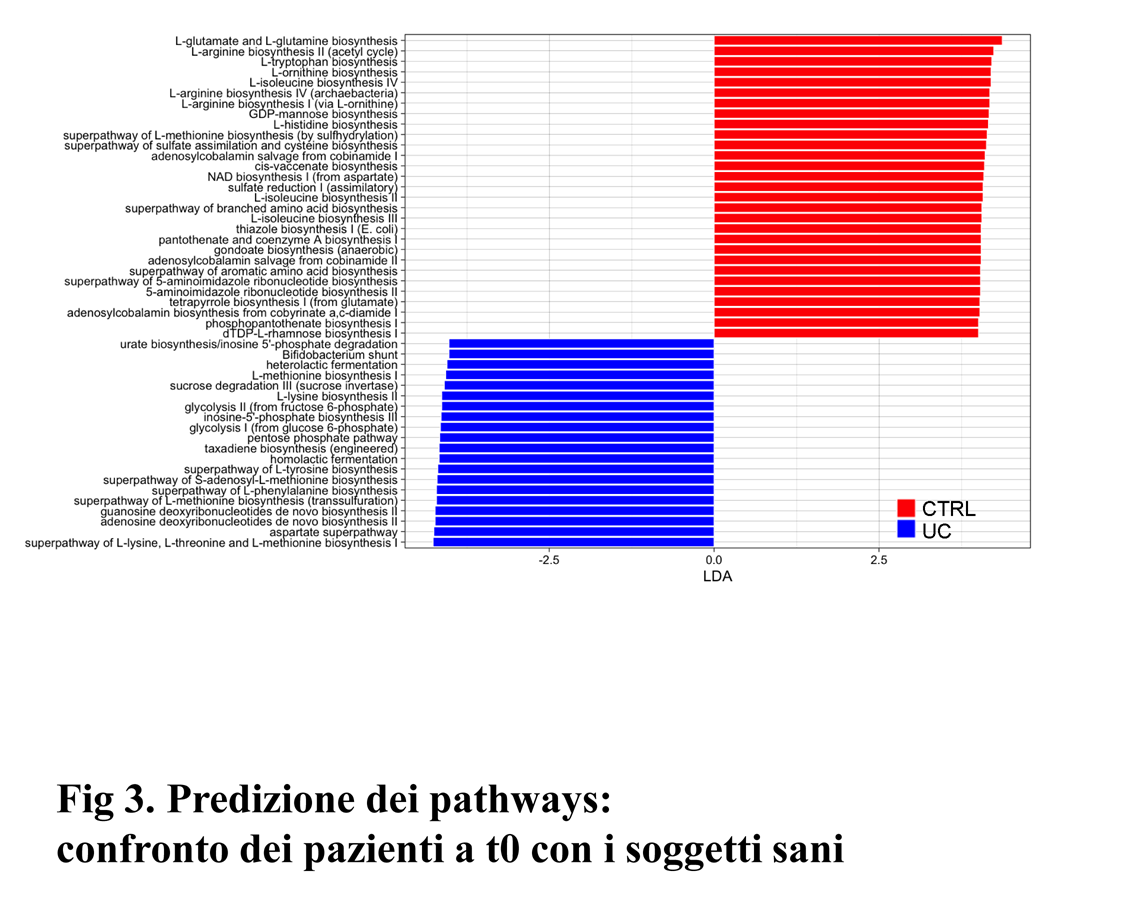


**Supplementary Figure 6.** Functional prediction using the MetaCyc database based on PICRUSt2. Twenty pathways, belonging to ten different metabolic classes, are associated to UC profile. Five pathways belonging to aminoacid biosynthesis were increased in UC such as superpathway of L-lysine, L-threonine and L-methionine biosynthesis I, superpathway of L-methionine biosynthesis (transsulfuration), superpathway of L-phenylalanine biosynthesis, superpathway of L-tyrosine biosynthesis, L-lysine biosynthesis II. An increase of aspartate superpathway was observed. About the carbohydrate degradation, the sucrose degradation III (sucrose invertase) pathway was increased. The superpathway of S-adenosyl-L-methionine biosynthesis is representative within the metabolic class called enzyme cofactor biosynthesis. In the fermentation to lactate class increase the homolactic and the heterolactic fermentation, in the fermentation to lactate/acetate increase *Bifidobacterium* shunt. Some pathways belonging to the generation of precursor metabolites and energy class such as pentose phosphate pathway, glycolysis I (from glucose 6-phosphate) and glycolysis II (from fructose 6-phosphate) and inosine-5'-phosphate biosynthesis III belonging to purine nucleotide biosynthesis were associated to UC profile. The plot shows the higher levels of adenosine deoxyribonucleotides de novo biosynthesis II and guanosine deoxyribonucleotides de novo biosynthesis II belonging to purine nucleotide de novo biosynthesis in UC respect with CTRL. Taxadiene biosynthesis (engineered) within the terpenoid biosynthesis is more representative in UC. There was also an increase of urate biosynthesis/inosine 5'-phosphate degradation in UC, whose metabolic class was not defined.

**
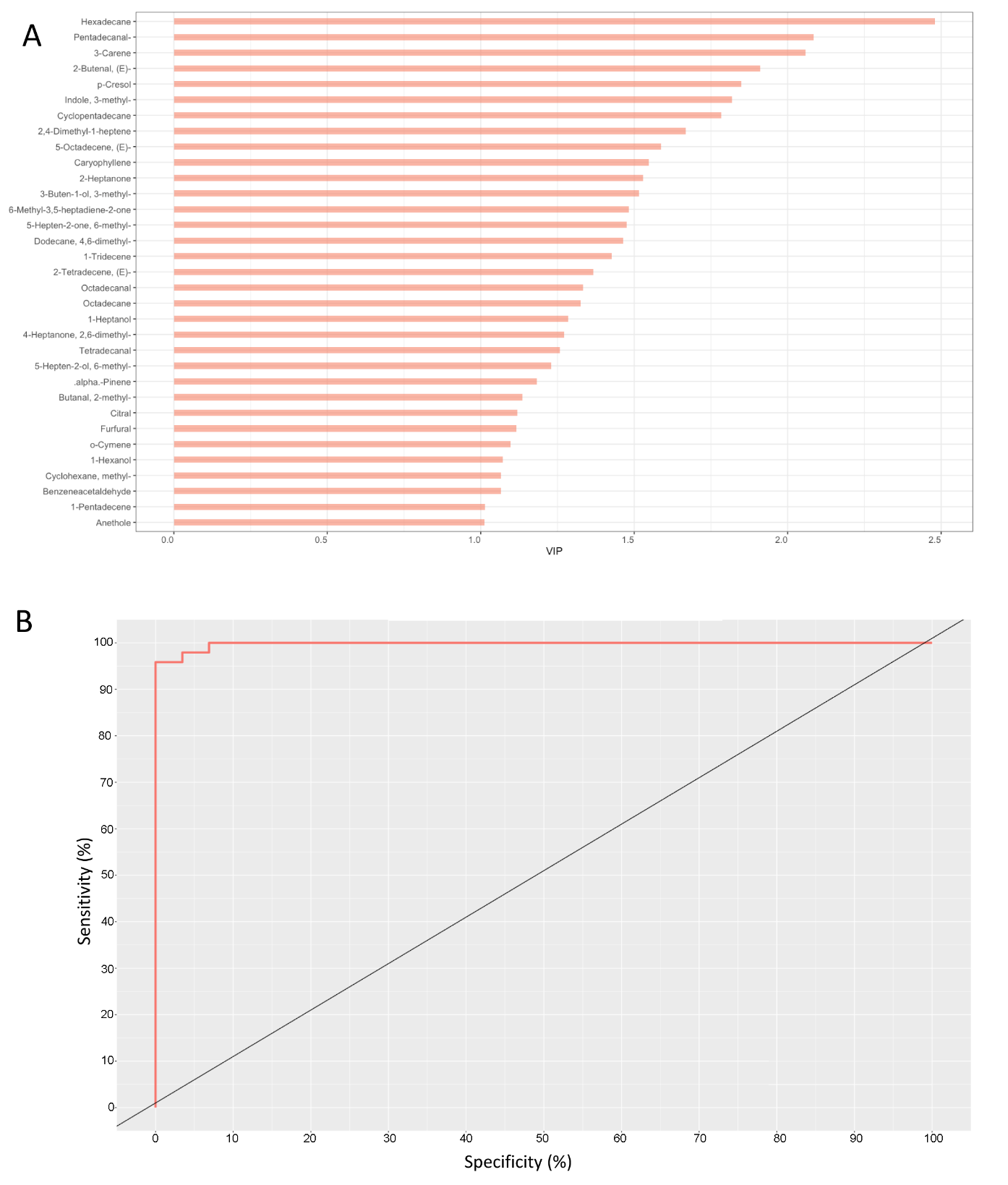
**

**Supplementary Figure 7.** Multivariate analysis on metabolic dataset. Bar plot of VIPs result from PLS-DA **(A)**. VIP values are reported on the horizontal axis. ROC analysis of the PLS-DA model **(B).** The value of AUROC = 0.9944 indicates a high accuracy of the prediction model.

## Supplementary Tables

**Supplementary Table 1.** Analysis of confounding factors. Comparison of the faecal microbiota, mycobiota and metabolome composition between subjects grouped by gender, age, and clinical features. Confounder function was applied to gender, median age (46 years), corticosteroid therapy, previous therapy and failure to previous therapies.

| **Characteristic** | ***p-*value** |
| --- | --- |
| Gender | 0.541 |
| Age (Median) | 0.032 |
| Corticosteroid therapy | 0.981 |
| Previous therapy | 0.301 |
| Failure to previous therapies | 0.785 |

**Supplementary Table 2.** Univariate analysis to investigate bacterial, fungal and metabolomic features associated to age. Linear discriminant analysis (LDA) Effect Size (LEfSe) was used to compare features of the gut microbiota of subjects grouped by median age (46 years). In this analysis, the FDR was used to assess the statistical significance.

| **Features** | ***p-*value** | **FDR** | **LDA score** | **direction** |
| --- | --- | --- | --- | --- |
| 3-Buten-1-ol, 3-methyl- | 0.0143 | 0.9289 | 4.21 | ≤ Median Age |
| Phenylethyl Alcohol | 0.0017 | 0.4782 | 4.17 | > Median Age |
| *Bulleidia* | 0.0280 | 0.9289 | 4.2 | > Median Age |
| *Haemophilus* | 0.0368 | 0.9289 | 4.21 | > Median Age |
| *Candida* | 0.0401 | 0.9289 | 4.16 | ≤ Median Age |
| *[Ruminococcus]* | 0.0130 | 0.9289 | 3.75 | > Median Age |
| *Blautia* | 0.0326 | 0.9289 | 4.28 | > Median Age |
| 2-Pentadecanone | 0.0212 | 0.9289 | 4.49 | > Median Age |

**Supplementary Table 3.** Network analysis between fungi and bacteria in UC and CTRL groups. The rho values of Spearman’s correlation, filtered for *p*-value <0.05, are reported.

| **UC** | | |
| --- | --- | --- |
| **Fungi** | **Bacteria** | ***rho* value** |
| *Rhizophydium* | *Haemophilus* | 0.6 |
| *Malassezia* | *Acinetobacter* | 0.51 |
| *Cladosporium* | *Acinetobacter* | 0.59 |
| *Sistotrema* | *Acinetobacter* | 0.58 |
| *Cryptococcus* | *[Prevotella]* | 0.6 |
| **CTRL** | | |
| **Fungi** | **Bacteria** | ***rho* value** |
| *Cryptococcus* | *Enterococcus* | 0.52 |
| *Malassezia* | *Eggerthella* | 0.52 |
| *Dioszegia* | *Eggerthella* | 0.58 |
| *Malassezia* | *Catenibacterium* | 0.58 |
| *Dioszegia* | *Catenibacterium* | 0.59 |
| *Malassezia* | LachnospiraceaeClostridium | 0.52 |
| *Dioszegia* | *Peptoniphilus* | 0.52 |
| *Pseudomicrostroma* | *Peptoniphilus* | 0.56 |
| *Fusariella* | *Peptoniphilus* | 0.59 |
| Capnodiales | *Peptoniphilus* | 0.56 |
| Basidiobolales | *Peptoniphilus* | 0.59 |
| *Hyaloscypha* | *Peptoniphilus* | 0.52 |
| Fungi | *Alistipes* | 0.55 |
| *Diplodia* | *Granulicatella* | 0.55 |
| *Trichophyton* | *Granulicatella* | 0.51 |
| Leotiomycetes | *Anaerococcus* | 0.56 |
| *Phlebia* | *Anaerococcus* | 0.52 |
| *Wallemia* | *Anaerococcus* | 0.56 |
| *Paraphaeosphaeria* | *Anaerococcus* | 0.52 |
| *Fusariella* | *Anaerococcus* | 0.59 |
| *Limonomyces* | *Anaerococcus* | 0.52 |
| Basidiobolales | *Anaerococcus* | 0.59 |
| Spizellomycetales | *Anaerococcus* | 0.52 |
| *Coniosporium* | *Anaerococcus* | 0.52 |
| *Malassezia* | ErysipelotrichaceaeClostridium | 0.51 |
| *Dioszegia* | *Haemophilus* | 0.51 |
| *Fusariella* | *Peptostreptococcus* | 0.68 |
| Basidiobolales | *Peptostreptococcus* | 0.68 |
| *Hyaloscypha* | *Peptostreptococcus* | 0.72 |
| *Saccharomyces* | *Parabacteroides* | 0.58 |
| *Hemimycena* | *Acidaminococcus* | 0.72 |
| *Peniophorella* | *Acidaminococcus* | 0.72 |
| Ascomycota | *Acidaminococcus* | 0.72 |
| *Sistotrema* | *Acidaminococcus* | 0.68 |
| *Malassezia* | *Parvimonas* | 0.55 |
| *Dioszegia* | *Parvimonas* | 0.56 |
| *Cryptococcus* | *Parvimonas* | 0.62 |
| *Trametes* | *Parvimonas* | 0.54 |
| *Coralloidiomyces* | *Parvimonas* | 0.51 |
| *Trichophyton* | *Parvimonas* | 0.56 |
| Rozellomycota | Rikenellaceae | 0.53 |
| *Malassezia* | *Acinetobacter* | 0.66 |
| *Dioszegia* | *Acinetobacter* | 0.59 |
| *Aureobasidium* | *Acinetobacter* | 0.51 |
| *Neosetophoma* | *Acinetobacter* | 0.52 |
| *Malassezia* | *Bulleidia* | 0.62 |
| *Dioszegia* | *Bulleidia* | 0.62 |
| *Aureobasidium* | *Bulleidia* | 0.52 |
| Laboulbeniomycetes | *Bulleidia* | 0.56 |
| *Xylodon* | *Bulleidia* | 0.67 |
| *Neosetophoma* | *Bulleidia* | 0.76 |
| *Cryptococcus* | *Bulleidia* | 0.55 |
| *Trichophyton* | *Bulleidia* | 0.55 |
| *Phlebia* | *[Prevotella]* | 0.68 |
| *Paraphaeosphaeria* | *[Prevotella]* | 0.68 |
| *Limonomyces* | *[Prevotella]* | 0.68 |
| Spizellomycetales | *[Prevotella]* | 0.68 |
| *Coniosporium* | *[Prevotella]* | 0.68 |
| *Malassezia* | *Prevotella* | 0.54 |
| Leotiomycetes | *Pseudoramibacter_Eubacterium* | 0.52 |
| *Wallemia* | *Pseudoramibacter_Eubacterium* | 0.52 |
| *Cutaneotrichosporon* | *Bacteroides* | -0.66 |
| *Vishniacozyma* | *Bacteroides* | -0.56 |
| *Wickerhamomyces* | *Bacteroides* | -0.55 |
| *Coniochaeta* | *Bacteroides* | -0.56 |
| *Fusicolla* | *Methanobrevibacter* | -0.58 |
| *Clavispora* | *Alistipes* | -0.53 |
| *Cutaneotrichosporon* | *Alistipes* | -0.7 |
| *Vishniacozyma* | *Alistipes* | -0.7 |
| *Wickerhamomyces* | *Alistipes* | -0.61 |
| Saccharomycetales | *Alistipes* | -0.6 |
| *Rhizophydium* | *Alistipes* | -0.51 |
| *Cutaneotrichosporon* | *Butyricicoccus* | -0.58 |
| *Wickerhamomyces* | *Butyricicoccus* | -0.52 |
| *Coniochaeta* | *Butyricicoccus* | -0.54 |
| *Clavispora* | *Sutterella* | -0.62 |
| *Cutaneotrichosporon* | *Sutterella* | -0.54 |
| *Candida* | *Sutterella* | -0.61 |
| *Vishniacozyma* | *Sutterella* | -0.61 |
| *Wickerhamomyces* | *Sutterella* | -0.52 |
| *Rhizophydium* | *Sutterella* | -0.52 |
| Saccharomycetales | *Prevotella* | -0.53 |
